# Comparative Study of Effects of Air-Entraining Plasticizing Admixture and Lime on Physical and Mechanical Properties of Masonry Mortars and Plasters

**DOI:** 10.3390/ma15072583

**Published:** 2022-03-31

**Authors:** Małgorzata Gołaszewska, Jacek Gołaszewski, Jerzy Bochen, Grzegorz Cygan

**Affiliations:** Faculty of Civil Engineering, Silesian University of Technology, ul. Akademicka 5, 44-100 Gliwice, Poland; jacek.golaszewski@polsl.pl (J.G.); jerzy.bochen@polsl.pl (J.B.); grzegorz.cygan@polsl.pl (G.C.)

**Keywords:** mortar, lime, aerating admixture, physical and mechanical properties

## Abstract

This article presents research on selected physical and mechanical properties of cement-based plasters and masonry mortars with consistency-improving additives, namely, traditional hydrated lime and a plasticizing and aerating mixture (APA), which, in practice, is often considered to be a lime substitute. Comparative analysis of the properties of mortars with alternative additives—lime or APA—was carried out, taking into consideration possible effects of cement, as two types of Portland cement were used for the research. For fresh mortar, mixture consistency, air content, resistance to segregation, and water retention were determined. Tests on hardened mortars included tests of porosity and impermeability, depth of penetration of water under pressure, drying shrinkage, as well as compressive and bending strength, modulus of elasticity, and adhesion of mortars to the base. In addition, research has shown that cement–lime mortars and cement mortars with APA admixture of similar consistency in the fresh state are characterized by significantly different properties. The results show, in most of the features analyzed, more favorable properties of mortars with the use of traditional lime. For shrinkage only, the use of admixture turned out to be more advantageous.

## 1. Introduction

In traditional construction technology, masonry and plastering mortars play an important role in erecting brick walls. Despite the ready-made system mortars, traditional mortars based on individual recipes are still used, for example, in renovation or restoration work, but also on smaller construction sites [1,2]. Historically, lime mortars were used; however, currently, cement mortars and cement–lime mortars are most popular in traditionally erected constructions [1]. In the literature, much attention was paid to the research of traditional mortars and their varieties, types of binder, additives, and admixtures and their influence on physical and mechanical properties [3] or durability [4]. Much mortar research concerns aggregate and fillers [5], and replacing cement with other materials, including waste [5,6,7,8,9,10], organic materials [11], and pozzolans [12,13].

An important and well-developed topic of traditional mortar research is the influence of technological factors such as the water-cement ratio [14], curing [15,16], temperature [16] and the way of dosing [17]. For example, the curing conditions were studied by Sajedi, who investigated different types of curing, including water, air, water-heated, oven-heated, air–water and water–air, finding that water curing and oven-heated curing had the best effects on the compressive strength of mortars [15]. In another study, higher strengths were achieved for ordinary Portland cement and ordinary Portland cement–slag mortars using lower binder content and a curing regime in water without heating, which found that curing in heated water decreases the compressive strength of ordinary Portland cement mortars [16]. As another example, Garijo et al. studied factors that have an effect on mortar properties, in particular the effects of dosage, water binder ratio, the mold material, aggregate size, and type and curing conditions [17].

Zhang et al. [18] presented a comparative study on the physical and mechanical properties and environmental resistance of natural and artificial hydraulic lime mortars. The results show that the mechanical properties of artificial mortars (cement-aerial lime-based CL and slag-aerial lime-based SL) are advantageous compared to those of natural hydraulic lime NHL mortar. In terms of environmental resistance, the SL mortar has been shown to have the best resistance to water and alkali, and CL mortar the best sulphate resistance. It should be noted, however, that while the research into the lime mortars is conducted on a regular basis in connection to the renovations and historical site studies [19,20], the research into lime as a constituent of cement–lime mortar is less popular [21].

A significant part of the research work concerns the modification of cement mortars with the addition of lime to improve their properties, with the simultaneous partial replacement of fillers or binders with other materials. For example, David [22], for the mix proportion 1:6, used 2%, 4% lime and 2%, 4% bamboo ash to partially replace the cement in the mortar. It was found that, with an increase in lime content, there is a significant reduction in strength, but in the presence of bamboo ash, the compressive strength of the mortar increased.

Many studies on the use of lime in mortars indicate its positive effect on mechanical and physical properties. Lime is a traditionally used additive that has a positive effect on the workability of mortars [23], their bonding strength, and microstructure [24,25].

In construction practice, lime is most well-known for its plasticizing effects, and is considered to be a plasticizing agent for cement–lime mortars.

However, due to the fact that cement–lime mortars have a longer setting time [26], with the development of admixtures, cement mortars became increasingly popular. Currently, methods for improving the properties of mortars are being developed using admixtures with different properties depending on the intended use [27,28,29]. They can be admixtures reducing the amount of water, plasticizing, liquefying, air-entraining, regulating binding and hardening, frost-resistant, affecting water adhesion or water resistance, or having a comprehensive effect [30,31]. For example, studies on the effectiveness of plasticizers and superplasticizers on the workability of mortars influenced by calcareous fly ash found that the presence of ash affects the efficiency of plasticizers and superplasticizers [27].

Admixtures with a plasticizing effect are frequently used, replacing the traditional addition of lime to cement-based mortars. In fact, many of the plasticizing admixtures are sold as ‘lime replacements’, due to the plasticizing effect of lime [32,33]. Therefore, assessing the differences in the use of both is important both from a theoretical and practical point of view, as it may affect the process of construction. Some researchers paid attention to plasticizing admixtures for mortars in their works [3,23,34]. Lenart [23] obtained results that showed that air-entraining and plasticizing admixtures improved workability but decreased compressive strength.

Another investigation [35] focused on the influence of traditional and non-traditional admixtures on mortar and concrete. The results showed that all the additives analyzed were plasticizing but the non-traditional admixtures had air-entraining effect. Due to this effect, the water absorbability of mortar and concrete increased and the strength decreased.

It should be noted that despite the interest in admixtures for masonry mortars and plasters, previous studies did not comprehensively compare properties of cement mortar with a plasticizing admixture and cement–lime mortar. Therefore, this paper aims to determine the differences in properties of plasters and masonry mortars with either lime addition, or air-entraining plasticizing admixture. The effect of lime and plasticizing admixtures on consistency could be similar [2], but this does not ensure similarity of their effects on properties of plastering and masonry mortars. In order to verify this assumption, a laboratory comparative analysis of the physical and mechanical properties of fresh and hardened cement mortars with the addition of lime and alternatively with an admixture replacing lime, having plasticizing and air-entraining properties, was carried out. To better judge the differences between the effect of lime and plasticizing admixture, the influence of cement and its origin were also analyzed.

## 2. Materials and Methods

### 2.1. Preparation of Mortars

Eight mortars were prepared for the investigation, four of which were plasters and the other four masonry mortars. In this amount, half of the mortars were made of cement–lime with a volume ratio of components: 1:1:6 (cement:lime:sand). The amount of water was selected to obtain the assumed consistency. The consistency of 9 cm of the Novikov’s cone was adopted for plastering mortars, 7 cm for masonry mortars. The other half of the mortars were prepared as cement mortars with a volume ratio of 1:6 (cement: sand), with the addition of an aerating plasticizing admixture (APA) replacing ordinary hydrated lime, acting as a plasticizer in its stead. This admixture is an aqueous solution of naphthalene resin and surfactants with a density of 1.040 ± 0.03 g/cm^3^, with an alkali content below 5% by weight and chlorides up to 0.1%. In the paste, the admixture surrounds the cement grains, giving them a homogeneous charge that causes their mutual repulsion, and thus plasticizes the cement mortar. Moreover, it improves cohesiveness, prevents segregation, and lowers the surface tension of the water, resulting in the formation of stable air micropores during mixing, which are regularly distributed throughout the volume of the mortar. The admixture content was assumed to be 0.25% of the cement mass, according to the manufacturer. The amount of water in these mortars was selected to obtain consistency as for mortars with lime. As a result, eight mortars for plastering and masonry were prepared, each with a different composition (Table 1). The mortars were made of CEM I 42.5 R Portland cement from two different producers, marked C1-G (CEM I 42.5 R NA) and C2-O (CEM I 42.5 R). The compositions of C1-G and C2-O are shown in Table 2, and their properties are shown in Table 3. Lime was used from one producer.

### 2.2. Testing Methods

Individual physical and mechanical properties were tested according to the following specific methods:

1. Consistency by the Novikov cone method was determined on a special device according to the standard PN-B-04500 [36]. A standard cone is mounted on the stand and lowered into the vessel with fresh mortar. After the mortar has been compacted and leveled, the cone is lowered, and the immersion depth is a measure of the consistency, which is determined as the average of the three tests.
2. The density of fresh mortar was determined according to the standard PN-EN 1015-6:2000 [37] using a cylindrical metal container with a diameter of 125 mm and a capacity of 1 dm^3^. After the vessel was filled with mortar and compacted, it was weighed. The density was determined as the average of two measurements based on the measured mass and volume of the mortar.
3. The air content in the mortar was determined with the pressure method according to the standard PN-EN 1015-7:2000 [38] using a cylindrical metal tank with a capacity of 1 dm^3^ with an air pressure chamber. After filling the tank with fresh air and calibrating the gauge, air is forced into the tank. The percentage of air blown in is a measure of the air content in the mortar. The result is the average of two consecutive measurements that differ by no more than 10%.
4. The segregation behavior of the mortar was determined according to the standard [36]. The test consists in measuring the change in mortar consistency in the upper and lower layer under the influence of vibration. The measure of susceptibility to segregation is the segregation index *K* defined by the formula:
  (1) $$K=\frac{\pi }{48}(S_{1}^{3}-S_{2}^{3})$$
  where: *S*_1_—measuring cone depression in the mortar from the upper layer (cm); *S*_2_—measuring cone depression in the mortar from the lower layer.
5. To determine water retention, a non-standardized procedure was used based on the guidelines of the standard PN-85/B-04500 [36], consisting of determining the amount of water withdrawn from the mortar and absorbed by the filter paper. The mortar was placed in the Vicat ring, which with a smaller diameter base was placed on the filter paper. The mortar sample was weighed before the *m*_o_ test and after 30 min of *m*_30_ water drainage through the paper layers. The water mass *m_w_* in the mortar placed in the Vicat ring was also determined. Mortar water retention was determined according to the following formula:
  (2) $$W=100-\frac{m_{o}-m_{30}}{m_{w}}\cdot 100\%$$
6. The total porosity and impermeability was determined for plasters, based on volume and real density. The volume density of hardened mortar was evaluated according to the water saturation test by masses weighed in three states: after drying in the temperature of 105 °C, at water saturation state and on a hydrostatical scale. The real densities of the mortars were determined in the Le Chatelier flask by measuring the weight and volume of the mortar crushed to the 0 0.063 mm fraction.
7. The apparatus was used to test the depth of penetration of water under pressure in mortar according to the PN-EN 12390-8: 2011 standard [39]. Cubic samples of mortars with a side of 150 mm were subjected to water pressure from the bottom side of 2 kPa for 60 min. After this time, the samples were split vertically, the depth of water penetration was determined, and the maximum, minimum, and mean values were calculated.
8. Mortar shrinkage was measured according to the standard PN-85/B-04500 [36] on a Graf Kaufman apparatus, for which the mortars were prepared in the form of 40 × 160 × 160 mm bars equipped with steel tips for measuring the shrinkage. The samples were installed in the apparatus in a vertical position, and at specified time intervals, after 3, 7, 14 and 28 days, readings of changes in the longitudinal dimension were made with an accuracy of 0.01 mm. The results were the mean of the three measurements.
9. The modulus of elasticity of the mortars was determined using the Pundit + concreteoscope. The dynamic modulus of elasticity was determined by the nondestructive ultrasonic method, which is the product of the bulk density of the mortar and the square of the ultrasonic impulse transit velocity through the mortar sample. The modulus was determined for three cubic samples 150 × 150 × 150 mm. The time it took for the ultrasonic wave to travel through the sample between the transmitting and receiving heads was measured, and the pulse velocity was determined on this basis.
10. The compressive strength tests of the half prismatic specimens were performed after testing of tension at flexural strength on a CONTROLS model 65-L27C12 strength test machine at a rate of loading of 2.4 kN/s for compression and 0.05 kN/s for bending. Features were tested in accordance with the standard [40]. During bending, the samples were supported on rollers with a diameter of 10 mm at a distance of 100 mm. Three samples were prepared for flexural testing and six for compressive testing.
11. The adhesion of the plasters to the base was determined according to the standard [36] by the ‘pull-off’ method of tearing previously glued metal discs with a diameter of 50 mm using a Pull-off tester DYNA Z-15 pull-off tester manufactured by Swiss Proceq-Schwitzerland with a range of 0–16 kN and a reading accuracy of 0.1 kN.

Each property tested was determined for a few specimens, no less than 3.

## 3. Results and Discussion

First, the properties of fresh mortars were compared, such as air content, delamination resistance, and water toughness. For hardened mortars, the following physical properties were determined: tightness and porosity, depth of penetration of water under pressure, and mortar shrinkage. The tested mechanical properties are: compressive and bending strength, modulus of elasticity, and adhesion of mortars to the base. The individual characteristics were compared with regard to cement origin and the type of binder additive—lime or lime substitute—which are factors that occur in construction practice when making mortars. The results obtained from the tested properties of cement mortars, mainly in the form after hardening, but also some characteristics of fresh mortars, show the effect of replacing the addition of lime with an APA admixture. In most of the characteristics analyzed, the replacement of lime with an admixture had an unfavorable effect.

### 3.1. Consistency and Air Content of Fresh Mortars

**Figure 1:**
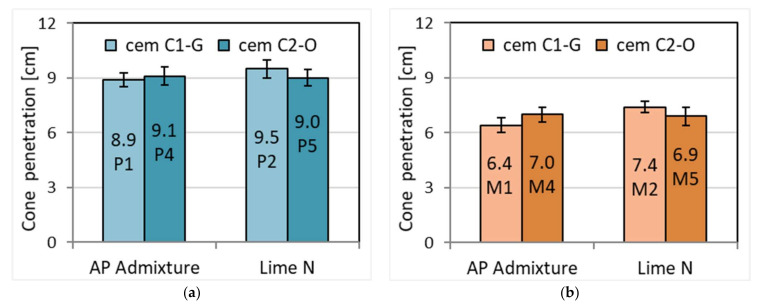
The effect of cement type and admixture type on the consistency of (**a**) plaster and (**b**) masonry mortar.

**Figure 2:**
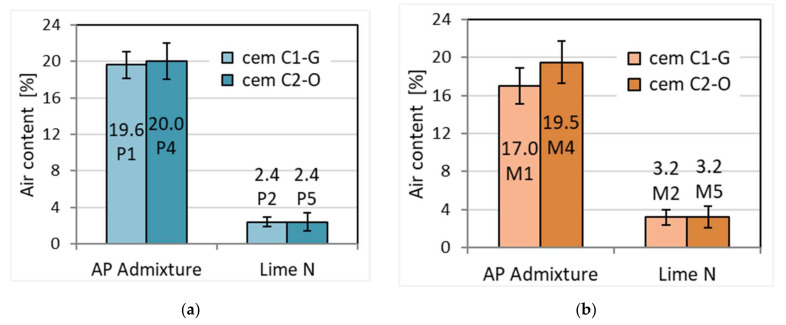
The effect of cement type and admixture type on the air content of (**a**) plaster and (**b**) masonry mortar.

For the prepared mortars, consistency was first checked. According to the initial assumption, the consistency of the masonry mortars had to be lower than that of the plaster mortar. This difference was obtained by adding the appropriate amount of water. The amount of water necessary for cement–lime was higher than in the case of cement mortar, as the water in cement–lime is well known to be higher than that in cement [41]. Therefore, similar cone fall values were obtained in the Novikov method, regardless of the origin of the cement and the cement binder additive (Figure 1). The addition of lime, with no change in water content, has been found to decrease consistency according to Quadir et al. [42], further confirming the obtained results. The plasticizing admixture, replacing lime, significantly decreased the density by about 15% to a value of 1800 kg/m^3^ compared to the density of over 2100 kg/m^3^ for mortars with the addition of lime (Table 4). The lower weight is due to the air-entraining effect of the admixture. The difference is quite significant considering that the admixture is only 0.25% by weight of cement and replaces lime in the amount of 50% by weight of cement. However, the density was not affected by the different origins of the cement. On the other hand, a clearly strong influence of the admixture was visible on the air content in fresh mortars, much more so than the addition of lime, which is related to differences in mortar density. The addition of APA admixture caused an approximately 6-fold increase in the amount of air to approximately 17–20%, while with the proportion of lime, it is approximately 3% (Figure 2). The high air content in the presence of APA admixture results from its air-entraining properties, which are stimulated by the foaming action of the admixture in the presence of water. This property translates into other properties of hardened mortars, e.g., lower tightness and greater porosity of hardened mortars, and lower water resistance, strength, and adhesion, as shown by further results.

### 3.2. Resistance to Segregation

**Figure 3:**
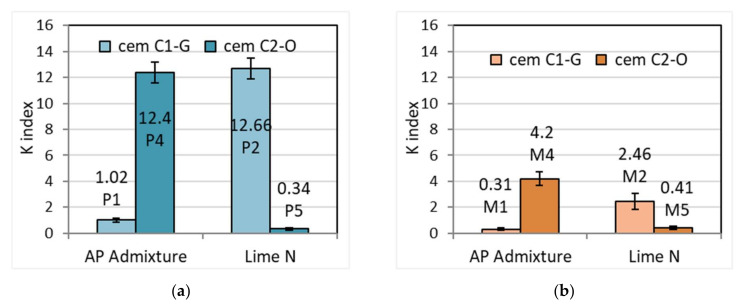
Index of susceptibility to segregation K for (**a**) plaster and (**b**) masonry mortars.

Plastering mortars, because of their more fluid consistency, are more susceptible to segregation. From a practical point of view, mortars with a greater segregation tendency must be mixed at the point of use. Interesting is the influence of the cement used on the K index of susceptibility to segregation, which determines the differences in the consistency of the mortar in the upper and lower layer after being subjected to vibration. The higher the value of the index, the greater the susceptibility to segregation. To determine the index in accordance with the standard methodology, the consistency measured by the Novikov cone was measured for both the upper and lower mortar layers, obtaining different values, both for plastering mortars and masonry mortars (Table 5). Masonry mortars have exhibited lower segregation susceptibility, possibly due to the lower amount of water and a stiffer structure, while plasters were less stable. The results of the tests show different values depending on the type of cement and the additive. In the case of the CEM I: C1-G cement, cement–lime mortars showed greater susceptibility to segregation, while the susceptibility to segregation of cement mortars with APA admixture turned out to be low (Figure 3). This effect was more noticeable in mortars of lower consistency. The opposite situation was observed for mortars with CEM I: C2-O cement. Cement–lime mortars showed less susceptibility to segregation compared to mortars with admixture. The effect was also more pronounced in mortars with higher consistency. This indicates different properties of cements despite their identical CEM I 42.5 R class. This indicates the possible issue of materials compatibility and the fact that the mortars should be checked before use if no problems arise with given materials combination.

### 3.3. Water Retention Tests

**Figure 4:**
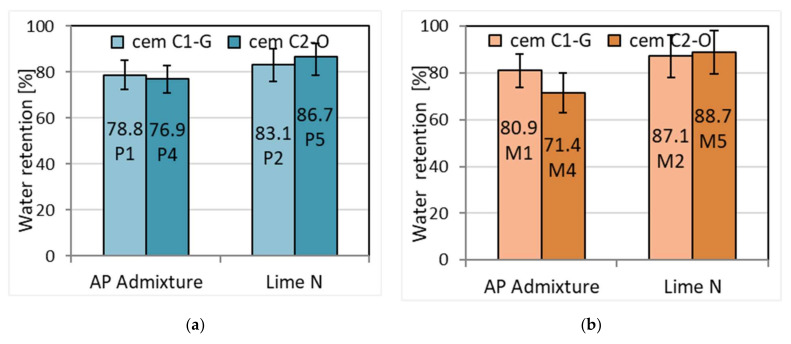
Water retention of (**a**) plaster and (**b**) masonry mortar.

Water retention, i.e., preventing water loss from the mortar, is an important parameter responsible for obtaining a number of important functional properties, e.g., shrinkage and adhesion to the surface. When masonry and plastering works, it is advisable to use mortars with higher water retention, especially when the mortars are used on materials with high absorbency. If there is rapid drainage of water from the mortar through the element of the wall, it may not be possible to correct its arrangement in the wall. Moreover, drainage of water that is too fast may have a negative impact on the adhesion of the mortar to the surface because then there are no proper conditions for creating strength and adhesion bonds. The results of the tests are presented in Table 6 and Figure 4. Comparing the obtained results, it can be concluded that cement–lime plaster and masonry mortars are characterized by an average of 12% higher water-retaining capacity than analogous cement mortars with APA admixture. The influence of the type of the cement was not observed, as the results for different cements turned out to be comparable. Similar results were obtained by Pavia and O’Brennan [43], who observed the water retention of cement–lime mortars to be 77–91%, and cement mortar to be 65–80%. Similarly, O’Looney et al. [43] compared cement mortars with lime and plasticizer, and water retention increased with lime content for all specimens.

### 3.4. Porosity and Impermeability of Mortars

**Figure 5:**
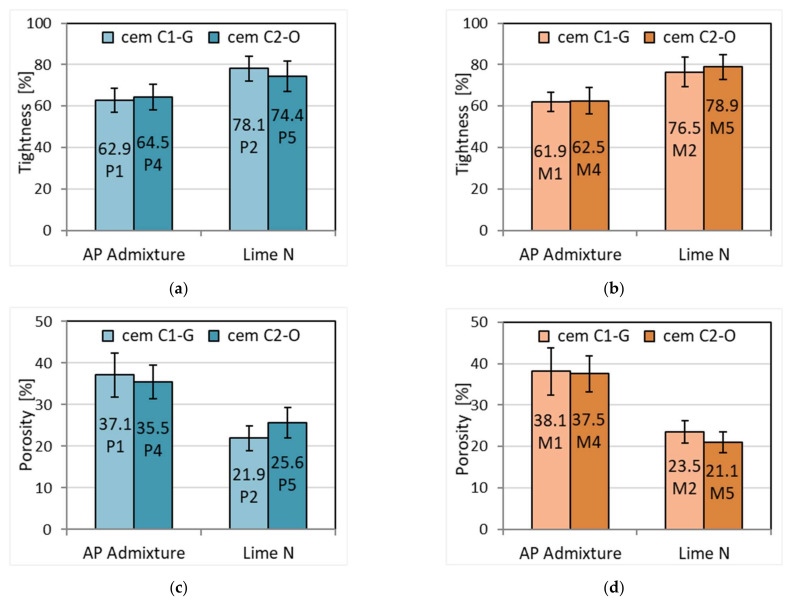
Impermeability and porosity of plasters (**a**,**c**) and masonry mortars (**b**,**d**).

The replacement of lime with plasticizing and air-entraining admixture clearly affects the tightness and porosity of the mortars. The tightness of cement–lime mortars is higher by an average of 22% (Figure 5, Table 7). This property translates into porosity, which for cement–lime mortars is about 21–25% and is lower than for cement mortars with APA admixture in the range of 35–38% (Figure 5), on average by 36%. It is undoubtedly connected with the higher air content in cement mortars with plasticizing and air-entraining admixture. No differences were observed due to the origin of the cement. In this respect, the results can be assessed comparably. The limiting effect of lime on porosity is related to its beneficial effect on the microstructure of the mortar due to the fine structure of lime grains below 5–7 μm in volume of almost 50%, which fill the larger pores. Therefore, the addition of lime reduces porosity and water permeability. Investigations by Silva et al. [44] found that cement–lime mortar shows a smaller porosity and lower water permeability compared to sand lime mortar. On the other hand, research by Marvila et al. [45] may lead to a conclusion that cement–lime mortars may have higher capillary porosity.

Lime also contributes to a greater amount of hydration products [18,46], which may also affect porosity.

### 3.5. Depth of Water Penetration under Pressure

**Figure 6:**
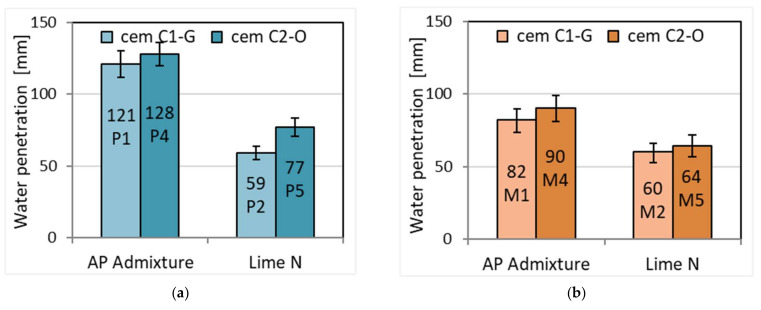
Depth of water penetration under pressure in (**a**) plasters and (**b**) masonry mortars.

**Figure 7:**
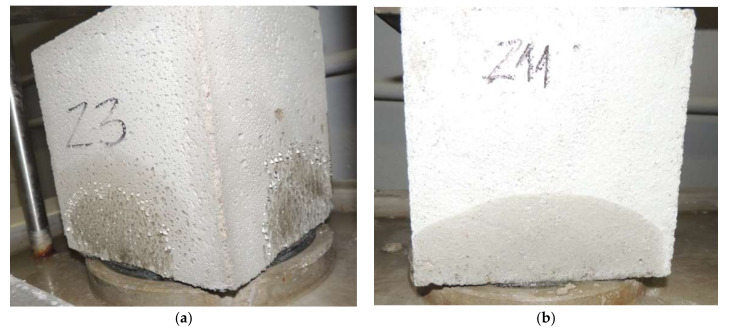
Example of tests of impermeability: (**a**) plaster M4; (**b**) masonry mortar M2.

**Figure 8:**
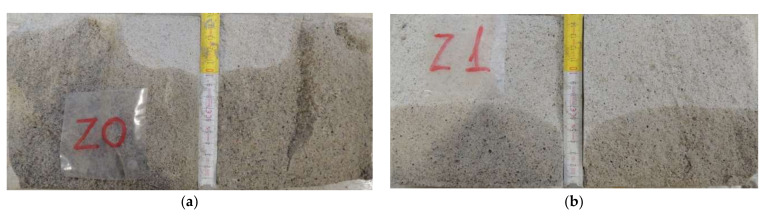

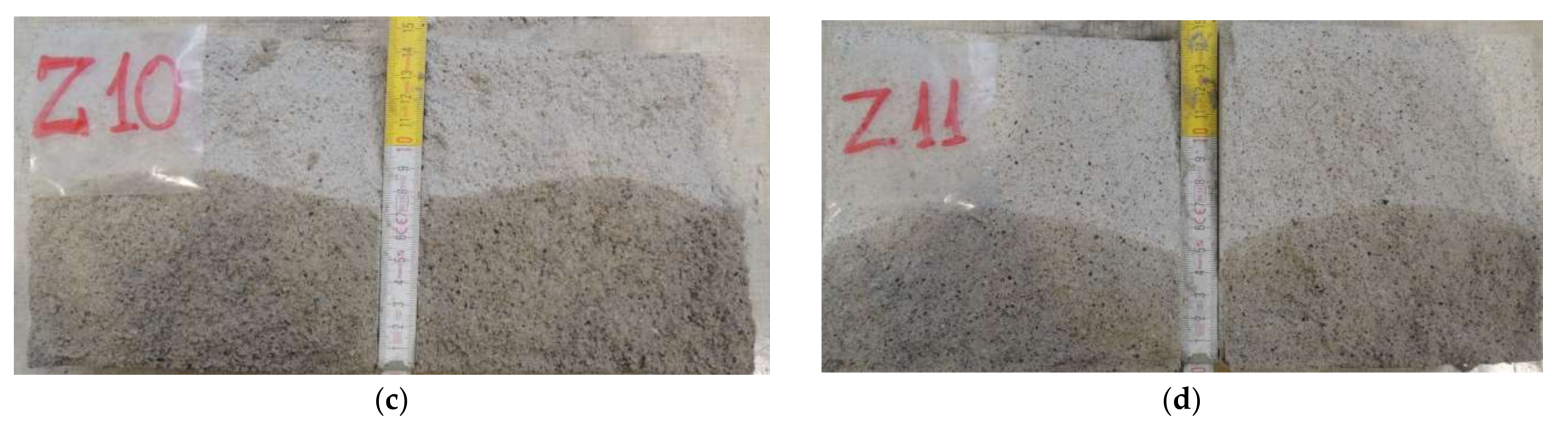
(**a**) Plaster with APA P1. (**b**) Plaster with lime P2. (**c**) Masonry mortar with APA M1. (**d**) Masonry mortar with lime M2. Depth of water penetration as seen and measured on chosen broken samples.

The mortar samples were subjected to a water pressure of 2 kPa for 60 min in an apparatus according to the standard method [39]. After this time, the samples were split and the minimum and maximum depth of water penetration was measured and the average value was determined (Table 8, Figure 6, Figure 7 and Figure 8).

The depth of penetration of pressurized water depends on the porosity of the mortar. The more porous the mortar, the greater the depth of water penetration. The depth of water penetration will be smaller the greater the proportion of closed pores in the porosity. The pore structure in this study has not been investigated. However, it is clearly visible that the water penetrates deeper into more porous cement mortars with APA admixture. Lime presence in cement mortar has been shown to increase porosity [45]; however, the effect of air-entraining admixture is stronger. The penetration depth is on average 86% higher for plaster mortars and 39% for masonry mortars. While the porous structure of mortars with APA is a deciding factor, it should be added that the addition of lime to cement–lime mortars has been found to reduce water permeability [47], and thus also water penetration under pressure. This difference could be even greater if there was no water condensation on the side surfaces of cement mortar samples with APA admixture (Figure 8). Water, finding a path with lower resistance, did not penetrate deeper into the sample. On the cement–lime mortar samples, only moisture was visible, but there was no water condensation (Figure 7). A slightly greater water penetration was observed for cement CEM C2-O in both types of mortars. It shows different properties of cements, probably with different proportions of ingredients.

### 3.6. Shrinkage of Mortars

**Figure 9:**
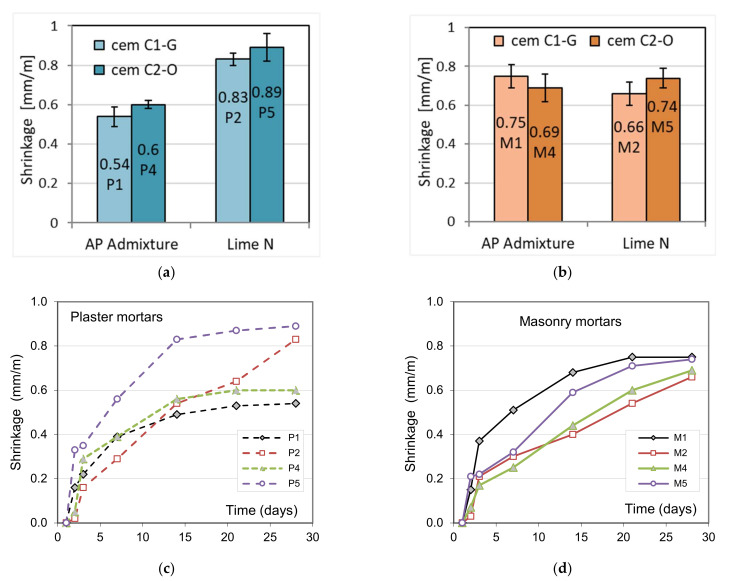
Shrinkage of plasters (**a**,**c**) and masonry mortars (**b**,**d**).

From the comparison of the results (Figure 9, Table 9), it was observed that the shrinkage during the drying of the plasters is lower in the case of cement mortars with APA admixture, on average by 33%. Thus, the beneficial effect of the admixture in reducing shrinkage can be seen. This effect should be associated with a lower *w*/*c* ratio of these mortars, having values 1.18 and 1.16, compared to mortars with lime with values of 1.53 and 1.38, respectively. The presence of air bubbles also has a positive effect, as confirmed by air content in fresh mortars, which can reduce the tensile stresses arising in the hardening cement slurry. This result is advantageous, particularly for plastering mortars, but also for masonry mortars. In the case of masonry mortars, the shrinkage of cement–lime and cement mortars with APA admixture after 28 days is at a similar level, which may result from lower consistency and lower water content, which is confirmed by the *w*/*c* index values (Table 1). The obtained results of the beneficial effect of the admixture on shrinkage confirm the work of other researchers. Lenart obtained the results [23] that the greatest reduction in shrinkage was observed for polymer admixtures (styrene–butadiene copolymer). The reduction in shrinkage occurred in the case of polyvinyl alcohol admixtures and the least plasticizing and air-entraining admixtures. The adverse effect of lime on shrinkage was observed by Sebaibi et al. [25]. They observed for a higher lime substitution of 10% the presence of microcracks in the matrix.

Jaafri et al. [48] ascertained that, in the context of shrinkage, lime addition to mortars can be treated as a filler, as it does not take part in cement hydration. Pavlík and Uzáková [49] found that the presence of mineral additions such as limestone powder, natural pozzolan, or slag at suitable cement replacement rates decreases drying shrinkage. These results are probably caused by the contribution of mineral additions to the production of other hydrates and a decrease in the volume of pores.

Similar results were obtained by Itim et al. [46], who found that the presence of mineral additions such as limestone powder, natural pozzolan, and slag at suitable cement replacement rates causes a decrease in drying shrinkage. These results are probably caused by the contribution of mineral additions to the production of other hydrates, which decreases the volume of pores.

### 3.7. Modulus of Elasticity and Strength of Mortars

**Figure 10:**
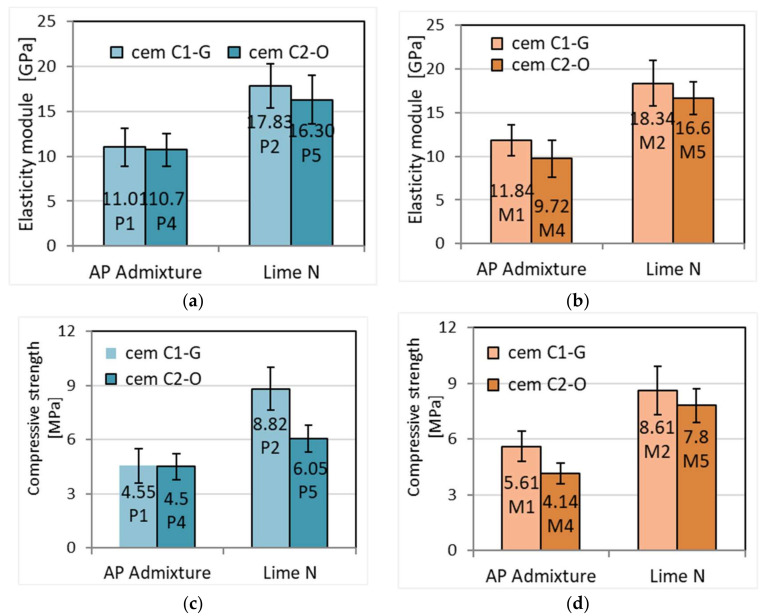
Dynamic elasticity modulus and compressive strength of plasters (**a**,**c**) and masonry mortars (**b**,**d**).

The results show that cement mortars with lime have a higher value of elasticity modulus, on average by 60%, compared to mortars with APA admixture (Figure 10). Differences in the dynamic modulus of elasticity are similar to differences in the compressive strength of mortars depending on the type of additive. Compressive strengths are lower with the use of APA admixture by about 40% compared to cement–lime mortars (Figure 10) (Table 10). In the tests presented, the proportion of lime in relation to cement is half of its mass, and the compressive strength reaches the value of 6–8.8 MPa.

The negative effect of the admixture on strength likely results from the air-entraining effect of the mortar, which was confirmed by tests of the air content in mortars and their porosity after hardening. The negative effect of APA admixture on strength is confirmed by the work of other researchers [23,34,50]. The addition of lime to cement mortars can lead to a decrease in compressive strength, even if porosity also decreases [51]. A similar relation, but with a smaller difference, applies to the bending strength Comparing the obtained results (Table 1 and Table 10), a decrease in strength for lower values of the water-cement index were also observed. Singh et al. [14] made a similar observation. In turn, Arandigoyen and Alvarez [24] observed that when 0–40% of cement is added to lime-based mortars, their mechanical strength increases slightly. In the case of cement mortars, the strength decreases sharply when a small amount of lime is added.

However, it should be added that lime could show the strengthening effect. The beneficial effect of small grain sizes below 5–7 μm, which constitute almost half of the overall volume, is that they fill the pore structure. The filler effect of small particles in mortar has been observed for many mineral additives. An example is the study by A. Itim et al. [48], in which the substitution of cement by 10%, 20% and 30% of limestone powder, natural pozzolan, and slag, respectively, involved an improvement in the compressive strength of the mortar. It is associated with a phenomenon of microstructure modification and additional hydrate production. It follows from this that the same effect can be obtained with lime.

**Figure 11:**
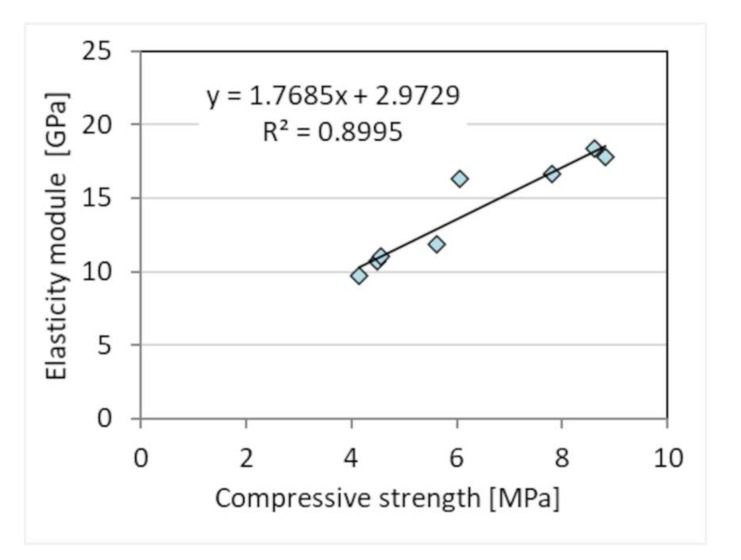
Relationship between dynamic elasticity modulus and compressive strength of mortars.

In the case of the modulus of elasticity, its value significantly depends on the strength, as evidenced by the linear regression with a high correlation R = 0.95 (Figure 11). Similar results of relation between compressive strength and dynamic elasticity modulus were obtained by [52].

The significant difference in the modulus of elasticity of cement–lime and admixture cement mortars makes them mortars of different deformability and means that they cannot be treated interchangeably. When selecting the mortar for masonry, its modulus of elasticity should be close to the modulus of elasticity of the joined elements. Then, the masonry structure will have the highest strength. The compatibility of the modulus of elasticity of mortars and connected elements becomes less important in the case of joining elements with thin 3 mm joints. The tested mortars are intended for traditional bricklaying with 10–12 mm thick joints; therefore, their different modulus of elasticity is important.

The reason for the reduction in modulus and strength can be found in the air-entraining properties of the admixture, resulting in a greater volume of air pores. This higher volume reduces the stress transfer surface. A similar effect can be found in the research of Arandigoyen and Alvarez [24]. The modulus of the elasticity of cement mortars decreased with the lime content. However, such mortars are more resistant to deformation and thus cracking.

### 3.8. Adhesion of Mortars to the Base

The adhesion of the tested mortars to the concrete and ceramic bases was determined. The concrete base consisted of 120 × 250 × 500 mm blocks of concrete with an aggregate size of 0–16 mm, *w*/*c* ratio = 0.5 and the amount of CEM I 42.5 R Portland cement equal to 350 kg/m^3^. The ceramic base was the base surfaces of standard class 100 building bricks. One concrete block and two bricks were prepared for each type of mortar. The blocks and bricks were prepared in a dry state and the surfaces were wetted three times with water before applying the mortars. After applying mortars with a thickness of 12 mm on the bases, the samples were stored for 28 days at a temperature of 20 °C and a relative air humidity of 50%. For each type of mortar, three or four pull-off measurements were made, and the result was determined as the arithmetic mean (Table 11).

**Figure 12:**
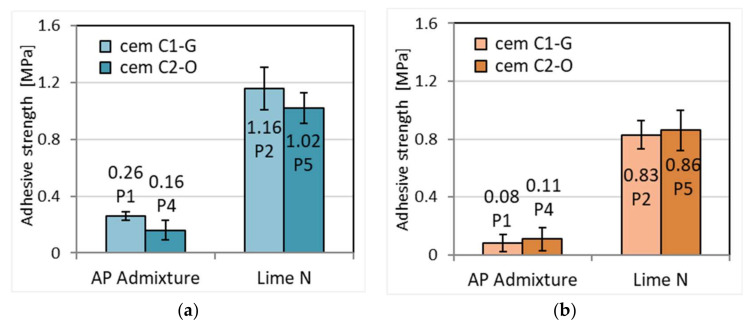
Adhesive strength to ceramic base of plasters (**a**) and masonry mortars (**b**).

**Figure 13:**
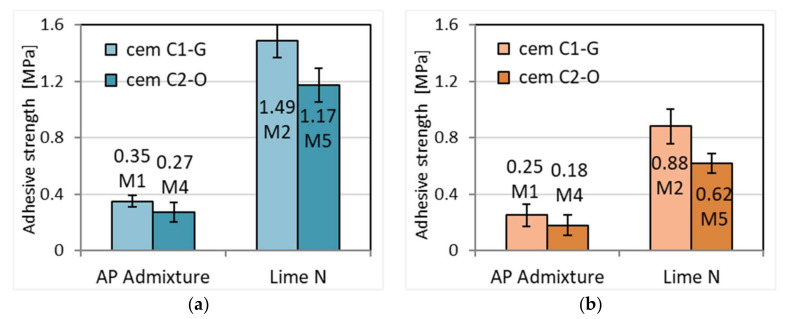
Adhesive strength of plasters (**a**) and masonry mortars (**b**) to concrete base.

The results showed a higher adhesion for cement–lime mortars for ceramic bases compared to mortars with APA admixture (Figure 12 and Figure 13). This effect is consistent with previous research on this topic [41,53,54]. Lime is considered to increase the retention of water in the mortar and therefore increase its bond strength. It prevents migration of water from the mortar to the base that is too rapid, making it available for longer for the binding processes of the binder. This is confirmed by the water permeability tests (Section 3.3). The increase in water retention in mortar can be explained by the significantly higher adhesion of cement–lime mortars to both bases than cement mortars with APA admixture. Greater adhesion of mortars to ceramic surfaces may result from their greater smoothness and lower porosity compared to concrete ones and thus lower water absorption, which improves the bond between the mortar and the base. Reduced adhesion of cement mortars with APA mixture is caused by high air content in the mortar, six times higher than in the mortar with the addition of lime (Section 3.1). Air bubbles are also present on the contact surface of the mortar and the base, which reduces its adhesion to the base. The origin of cement has little effect on adhesion. In three out of four cases, the C1-G cement had a slightly better effect.

## 4. Conclusions

Tests of cement–lime mortars and cement mortars with APA admixture intended for plastering or masonry mortars of similar consistency in the fresh state show that the properties of these mortars differ significantly from each other. On the basis of the research, it can be concluded that:

1. The segregation susceptibility of the tested mortars depends on the type of cement used. For Cem 1-G mortars, cement–lime mortars are more susceptible to delamination, while the susceptibility to delamination of cement mortars with APA admixture is negligible. The opposite is true for Cem 2-O cement mortars, as cement–lime mortars are less susceptible to delamination.
2. Cement–lime mortars, both plasters and masonry mortars, regardless of the type of cement, are characterized by an average of 12% higher water-retaining capacity than analogous cement mortars with APA admixture.
3. The air content in fresh mortars with APA admixture is six times higher than in mortars with the addition of lime. This results in lower porosity of cement–lime mortars than cement mortars with APA admixture by an average of 38%, and their impermeability is higher by an average of 22%.
4. The resistance to water penetration of cement–lime mortars is greater than that of analogous cement mortars with APA admixture, which is a consequence of greater tightness and lower porosity.
5. Drying shrinkage of plasters is lower in the case of cement mortars with APA admixture. In the case of masonry mortars, the shrinkage of cement–lime and cement mortars with APA admixture after 28 days is at a similar level.
6. The modulus of elasticity of cement–lime mortars is greater than the modulus of elasticity of analogous cement mortars with APA admixture, which reflects different strengths.
7. Cement–lime mortars, regardless of the type of base and type of cement used, are characterized by greater adhesion to the base than analogous cement mortars with APA admixture.

To sum up, mortars of similar workability, however, obtained by different means—one using admixture, the other using lime—show different properties. The use of APA ensures the appropriate consistency of the mortar by airing it. However, this increases the porosity of the mortar and reduces its water impermeability. Due to its large specific surface, lime has the ability to retain water, so the workability improvement effect occurs without the air-entraining effect characteristic of APA admixtures. Consequently, cement mortars with APA admixture, compared to cement–lime mortars, are characterized by significantly lower mechanical properties (compressive strength, modulus of elasticity), lower water resistance, and, moreover, worse adhesion to the base.

From a practical standpoint, the research results presented in this paper allow for a better understanding of the possible effects of replacing lime with APA and vice versa, which can, in turn, allow one to better choose the right plasticizing agent.

In conclusion, the use of plasticizing and air-entraining admixture as a lime substitute requires caution because, despite the expected effect on the consistency of the fresh mortar, it adversely affects all mechanical and almost all physical properties. The exception is mortar shrinkage, which is smaller in the presence of admixture. The choice of using an admixture instead of adding lime should be made with this effect on properties in mind.

## Figures and Tables

**Table 1:** Composition of plaster (P) and masonry mortars (M) (kg/m^3^).

Constituent (kg)	Plaster (P)				Masonry Mortars (M)			
	P1	P2	P4	P5	M1	M2	M4	M5
C1-G: CEM I 42.5 R NA	169	186	-	-	177	189	-	-
C2-O: CEM I 42.5 R	-	-	165	189	-	-	171	192
APA.	0.426	-	0.426	-	0.446	-	0.446	-
Lime	-	89	-	91	-	91	-	92
Sand	1455	1599	1417	1629	1525	1625	1473	1649
Water	197	284	195	260	189	271	188	250
Water-binder ratio	1.16	1.03	1.18	0.93	1.07	0.97	1.10	0.88
Water-cement ratio	1.16	1.53	1.18	1.38	1.07	1.43	1.10	1.30

**Table 2:** Composition of cements C1 and C2.

	Phase Composition [%]				Oxide Composition [%]										
	C_3_S	C_2_S	C_3_A	C_4_AF	SiO_2_	Al_2_O_3_	Fe_2_O_3_	CaO	MgO	Na_2_O	K_2_O	Na_2_O_eq_	SO_3_	Cl	LOI
**C1**	62.4	12.2	7.6	8.5	20.6	4.67	2.8	64.4	1.2	0.18	0.4	0.46	2.79	0	2.8
**C2**	60.7	11.6	11.9	8.2	19.9	6.2	2.7	62.6	1.5	0.33	0.7	0.8	2.6	0.1	2.9

**Table 3:** Basic properties of cements.

Cement Property	Unit	Value for	
		C1	C2
Initial setting time	min	167	196
Soundness of cement, by Le Chatelier’s method	mm	0.4	0.3
Flexural strength after 2 days	MPa	7.3	6.7
Compressive strength after 28 days	MPa	56.8	44.8
Specific surface area	cm^2^/g	4400	4390

**Table 4:** Consistency and air content of the tested mortars.

Constituents (kg)	Plaster Mortar				Masonry Mortar			
	P1	P2	P4	P5	M1	M2	M4	M5
Density [kg/m^3^]	1821	2158	1778	2169	1892	2176	1832	2183
Cone penetration [cm]	8.9	9.5	9.1	9.0	6.4	7.4	7.0	6.9
Air content A_c_ [%]	19.6	2.4	20.0	2.4	17.0	3.2	19.5	3.2

**Table 5:** Resistance to segregation test results.

Property	Plaster				Masonry Mortar			
	P1	P2	P4	P5	M1	M2	M4	M5
Index of susceptibility to segregation K [-]	1.02	12.66	12.40	0.34	0.31	2.46	4.20	0.41

**Table 6:** Results of water retention tests.

Property	Plaster				Masonry Mortar			
	P1	P2	P4	P5	M1	M2	M4	M5
Mass of Vicat ring with mortar m_0_ [g]	310.6	382.1	310.8	377.8	329.0	380.4	322.3	384.8
Mass of Vicat ring with mortar after 30 min m_30_ [g]	303.5	373.6	302.9	371.8	322.6	374.3	313.1	379.8
Amount of water put into Vicat ring m_w_ [g]	33.5	50.2	34.1	45.3	33.5	47.4	32.1	44.1
Water retention W [%]	78.8	83.1	76.9	86.7	80.9	87.1	71.4	88.7

**Table 7:** Porosity and impermeability of mortars.

Property	Plaster				Masonry Mortar			
	P1	P2	P4	P5	M1	M2	M4	M5
Density of hardened mortar after drying, [kg/dm^3^]	1.650	1.952	1.640	1.937	1.632	1.957	1.626	1.982
Specific density, [kg/dm^3^]	2.623	2.499	2.543	2.603	2.637	2.558	2.602	2.512
Impermeability, [%]	62.9	78.1	64.5	74.4	61.9	76.5	62.5	78.9
Porosity, [%]	37.1	21.9	35.5	25.6	38.1	23.5	37.5	21.1

**Table 8:** Depth of water penetration under pressure.

Property	Plaster				Masonry Mortar			
	P1	P2	P4	P5	M1	M2	M4	M5
Minimal depth of water penetration H_min_ [mm]	97	46	145	65	74	48	70	54
Maximal depth of water penetration H_max_ [mm]	145	71	111	88	90	71	110	74
Average depth of water penetration H_średn_ [mm]	121	59	128	77	82	60	90	64

**Table 9:** Shrinkage of plasters and masonry mortars.

Time [Days]	Drying Shrinkage [mm/m]							
	P1	P2	P4	P5	M1	M2	M4	M5
1	0.00	0.00	0.00	0.00	0.00	0.00	0.00	0.00
2	0.16	0.02	0.05	0.33	0.15	0.03	0.07	0.21
3	0.22	0.16	0.29	0.35	0.37	0.21	0.17	0.22
7	0.39	0.29	0.39	0.56	0.51	0.30	0.25	0.32
14	0.49	0.54	0.56	0.83	0.68	0.40	0.44	0.59
21	0.53	0.64	0.60	0.87	0.75	0.54	0.60	0.71
28	0.54	0.83	0.60	0.89	0.75	0.66	0.69	0.74

**Table 10:** Strength and elasticity modulus of mortars.

Property	Plaster				Masonry Mortar			
	P1	P2	P4	P5	M1	M2	M4	M5
Apparent density [kg/dm^3^]	1.654	1.957	1.648	1.946	1.640	1.970	1.631	1.989
Impulse transit time [μs]	58.13	49.70	58.87	51.90	55.83	49.17	61.47	51.87
Impulse velocity [m/s]	2584.4	3018.2	2548.2	2892.0	2686.7	3051.2	2441.8	2892.1
Dynamic elasticity modulus E_dyn_ [GPa]	11.01	17.83	10.70	16.28	11.84	18.34	9.72	16.63
Compressive strength [MPa]	4.55	8.82	4.50	6.05	5.61	8.61	4.14	7.80
Tensile strength [MPa]	2.2	3.0	1.3	1.4	12.4	2.7	1.9	2.3

**Table 11:** Adhesion of mortars to base.

Property	Plaster				Masonry Mortar			
	P1	P2	P4	P5	M1	M2	M4	M5
Adhesive strength to ceramic base [MPa]	0.26	1.16	0.16	1.02	0.35	1.49	0.27	1.17
Adhesive strength to concrete base [MPa]	0.08	0.83	0.11	0.86	0.25	0.88	0.18	0.62

## Data Availability

Data sharing not applicable.
